# Complement in Metastasis: A Comp in the Camp

**DOI:** 10.3389/fimmu.2019.00669

**Published:** 2019-04-03

**Authors:** Daniel Ajona, Sergio Ortiz-Espinosa, Ruben Pio, Fernando Lecanda

**Affiliations:** ^1^Program in Solid Tumors, Center for Applied Medical Research (CIMA), University of Navarra, Pamplona, Spain; ^2^Navarra Institute for Health Research (IdISNA), Pamplona, Spain; ^3^Centro de Investigación Biomédica en Red de Cáncer (CIBERONC), Madrid, Spain; ^4^Department of Biochemistry and Genetics, School of Sciences, University of Navarra, Pamplona, Spain; ^5^Department of Pathology, Anatomy and Physiology, School of Medicine, University of Navarra, Pamplona, Spain

**Keywords:** cancer, metastasis, complement, tumor microenvironment, anaphylatoxin, bone colonization

## Abstract

The complement system represents a pillar of the innate immune response. This system, critical for host defense against pathogens, encompasses more than 50 soluble, and membrane-bound proteins. Emerging evidence underscores its clinical relevance in tumor progression and its role in metastasis, one of the hallmarks of cancer. The multistep process of metastasis entails the acquisition of advantageous functions required for the formation of secondary tumors. Thus, targeting components of the complement system could impact not only on tumor initiation but also on several crucial steps along tumor dissemination. This novel vulnerability could be concomitantly exploited with current strategies overcoming tumor-mediated immunosuppression to provide a substantial clinical benefit in the treatment of metastatic disease. In this review, we offer a *tour d'horizon* on recent advances in this area and their prospective potential for cancer treatment.

## Introduction

The complement system represents a master component effector of innate immunity. Complement activation and regulation encompasses more than 50 soluble and membrane-bound proteins.

The function of complement, which entails the recognition and removal of pathogens and harmful entities, is accomplished by a multistep and sequential serine proteases-mediated cascade. The release of proteolytic fragments mediates key homeostatic and effector functions including: opsonization, inflammation, adaptive immune regulation, coagulation, tissue repair, neural development, bone homeostasis, angiogenesis, and host–microbiota symbiosis (1). Owing to the potentially deleterious effects of the complement system, its activity is tightly regulated at different levels by a number of soluble and membrane-bound proteins (2). Inappropriate complement activation underlies a variety of physiopathological conditions including inflammatory diseases and cancer (3).

Because many of the complement functions modulate tumor progression, their preeminent roles in promoting tumor cell dissemination are not surprising. This review focuses on recent findings on the major role of the complement system in tumor progression and highlights its key contribution to the different steps of the metastatic cascade.

## Complement Activation

Complement is mainly activated via three different recognition pathways: the classical, the lectin, and the alternative pathways. These three modes of complement activation converge into the generation of C3 convertases, which cleave C3 into C3a and C3b. C3a is an anaphylatoxin displaying an inflammatory regulation role. C3b can act in the opsonization process and as a component of the C5 convertase (4).

The classical pathway is triggered by the binding of C1q to antigen-antibody complexes, dying cells, extracellular matrix proteins, pentraxins, amyloid deposits, prions, or DNA (5).

The lectin pathway starts through binding of proteins homologous to C1q (mannose-binding lectin and H-, L-, or M-ficolins) to carbohydrate structures on pathogens (6). Both the classical and the lectin pathways then sequentially cleave C4 and C2 for the generation of the classical/lectin C3 convertase (C4bC2b) (4).

Finally, the alternative pathway is initiated by the spontaneous hydrolysis of C3, also known as the “tickover” of C3, which after the formation of C3(H_2_O) can bind to factor B. Cleavage of factor B by factor D forms the initial alternative pathway C3 convertase, C3(H_2_O)Bb (7).

Although these three routes of activation differ in their mechanisms of target recognition and initiation, they converge at C3 cleavage, yielding the active fragments C3a and C3b. C3b binding to C3 convertases assembles the C5 convertase that cleaves C5 into the anaphylatoxin C5a, and C5b. The latter fragment is indispensable to assemble the membrane attack complex which mediates targeted lysis (8).

Additional pathways of complement activation include C3 and C5 extrinsic protease cleavage (9–11), the C2-bypass pathway (12), and the properdin-mediated direct convertase formation on microbial surfaces (13).

Among the complement-derived downstream effectors, C3a and C5a play diverse roles in both homeostasis and disease. These molecules bind to their cognate seven-transmembrane domain receptors C5a receptor 1 (C5aR1; CD88) and C3a receptor (C3aR), respectively. C5a can also bind to C5aR2 (14). The role of C5aR2 remains poorly understood. Recently, it has been reported that the binding of C5a to C5aR2 in carcinoma-associated fibroblasts promotes tumor formation and chemoresistance by providing a survival niche for cancer stem cells (15).

Recent discoveries have also revealed that complement activation is not only restricted to the extracellular space, as originally thought, but also occurs in the cytoplasm. The intracellular components of complement (the so-called complosome) modulate metabolic processes during T cell effector differentiation (16, 17) but so far, their intracellular functions remain largely unexplored.

## Complement in Cancer Patients

Neoplastic transformation involves complex genomic and epigenomic alterations perturbing normal cell homeostasis. Local or distant dissemination of tumor cells, one of the hallmarks of cancer, represents a multistep process that entails the gain of novel cellular functions which include invasion, increased cell locomotion, intravasation, survival in the circulation, overcoming immune attack, and colonization in foreign cellular niches to form secondary tumors (18).

Overcoming immune attack is a key step in tumor progression. Altered immune recognition is achieved by a variety of mechanisms (19), including the modulation of the complement system. Complement activation has been described in cancer patients with hematological malignancies such as lymphomas (20), and in a plethora of solid tumors (21–23). Furthermore, intact complement proteins were found increased in blood of patients with lung cancer (24, 25), neuroblastoma (26), and digestive tract tumors (27). However, complement-mediated cytotoxicity is circumvented by different mechanisms, most of which include the upregulation of complement regulatory proteins (28–30). These regulators normally protect tumor cells from complement-mediated destruction, and can be grouped into two categories: membrane-bound complement regulatory proteins (mCRPs) and soluble regulators. High expression of the mCRPs membrane cofactor protein (CD46), decay-accelerating factor (CD55), and CD59 (protectin) on tumor cells is associated with increased metastatic potential, and poor prognosis in a range of tumors (31–34). Similarly, the soluble regulators factor H and FHL-1 have been found elevated in biological fluids from ovarian (35), bladder (36) and lung cancer patients (37), and are also associated with poor prognosis (38). Other soluble regulators as clusterin (39), C1 inhibitor (40), factor I and C4b-binding protein (C4BP) (41) are secreted by tumor cells into the tumor milieu and could also be detected in the circulation.

Activation of the complement system by tumor cells was long believed to only benefit the patient. Preclinical data suggest that complement can evoke potent complement-dependent cytotoxicity against tumor cells, and a range of therapeutic strategies have been designed to potentiate complement activation and overcome the protection mediated by complement inhibitors. This approach has been specially tailored to enhance the therapeutic efficacy of monoclonal antibodies (42). However, recent findings have challenged this view, providing evidence of the cancer-promoting potential of complement activation and the utility of complement inhibition as an anticancer therapy (43). Complement components coopted by tumor cells can lead to the acquisition of self-advantageous functions tilting the balance toward tumor progression. For instance, lung cancer cells are recognized by the complement system more efficiently than their normal counterparts. This effect is mediated by the direct binding of C1q and leads to the subsequent activation of the classical complement pathway (44). This activation is compensated by the expression of factor H/FHL-1 and CD59 (45, 46). This equilibrium in complement activity would explain the elevated levels of complement fragments found in biological fluids from these patients. Thus, C4d, a split product of the classical complement pathway, is increased in biological fluids of lung cancer patients. Detection of C4d is associated with poor prognosis, and has been proposed as a potential biomarker of clinical value in the management of lung cancer patients (44, 47, 48). Similar results were obtained in oropharyngeal tumors by detecting C4d in saliva (49). Moreover, other complement factors have been associated with cancer. Anaphylatoxin C5a is increased in plasma from lung cancer patients (50, 51), and is associated with metastatic potential in lung and gastric cancer patients (52, 53). Similarly, C1QB is one of the top-scoring genes associated with lung metastases in osteosarcoma patients (54).

Taken together, these studies indicate an association between complement activation and malignant progression.

## Complement in the Tumor Microenvironment

Of all complement proteolytic fragments derived from complement activation, anaphylatoxins are by far, the best described in cancer. Anaphylatoxins C5a and C3a trigger spurious tumor intracellular signaling pathways by binding to their cognate receptors expressed in tumor and immune cells. These signaling events deeply perturb the tumor milieu by inducing the recruitment and/or tumor-promoting abilities of myeloid-derived suppressor cells (MDSC), macrophages, neutrophils, and mast cells, preventing efficient T cell-mediated responses (55).

Elevation of C5a or C5aR1 levels has been observed in solid tumors including lung (50, 53), gastric (56), ovarian (57), breast (58), urothelial (59), and clear cell renal cancers (60).

C5a induces the recruitment of MDSCs into the tumor microenvironment, and markedly dampens anti-tumor T-cell responses. C5aR1 mediates these effects on two subpopulations of MDSCs. On one side, C5a is a potent chemoattractant for granulocytic MDSCs (a neutrophil-like subpopulation) and on the other, C5a stimulates the monocytic MDSC subpopulation with the concomitant production of reactive oxygen and nitrogen species (61).

C5aR1 expressed on MDSCs is also able to bind ribosomal protein S19 (RPS19), which is released from apoptotic tumor cells into the tumor microenvironment, leading to a shift toward Th2 cell responses with increased levels of immunosuppressive TGF-β (62). Accordingly, pharmacological blockade of C5aR1 in a syngeneic model of lung cancer impaired tumor growth, decreased the percentage of splenic MDSCs, and downregulated immunosuppression-related genes including ARG1, IL6, IL10, CTLA4, LAG3, and PDL1 within the tumor milieu (50).

Besides MDSCs, C5a affects the biology of other leukocytes present in the tumor microenvironment. C5a elicits a strong pro-inflammatory infiltration with secretion of MCP-1, responsible for the recruitment of immunosuppressive macrophages, and increase of arginase-1 and IL-10 (63). Similarly, fibrinolytic enzyme-mediated generation of C5a regulates the protumorogenic properties of C5aR1^+^ mast cells and macrophages, leading to hampered antitumor CD8 T-cell responses in a model of squamous carcinogenesis. Interestingly, the combined treatment based on cytotoxic chemotherapy and the blockade of C5aR1 synergistically increased the recruitment and the cytotoxic properties of CXCR3^+^ effector memory CD8 T cells by IFNγ-dependent mechanisms (64). Ablation of PTX3, an important negative regulator of inflammation and complement activation, resulted in amplification of complement activation, MCP-1 production, and tumor-promoting macrophage recruitment. Consistently, pharmacological blockade of C5aR1 reversed these pro-tumorogenic effects (65).

Although far less studied than C5a, the anaphylatoxin C3a also preconditions a tumor-promoting microenvironment. Signaling mediated by C3a binding to C3aR contributes to melanoma tumorigenesis by inhibiting neutrophil and CD4 T-cell responses (66). Autocrine complement C3 inhibits IL-10-mediated cytotoxic properties of tumor-infiltrating CD8 T lymphocytes through complement receptors C3aR and C5aR1, and enhances melanoma and breast cancer growth (67).

Moreover, complement activation may underlie the ability of tumors to evolve and adapt to different cues of the microenvironment increasing tumor progression. Thus, under hypoxic conditions, lung cancer cells downregulate complement inhibitors, factor H and factor I, to increase their susceptibility to complement activation (68). This phenomenon may fuel the generation of C5a which in turns may contribute to hypoxic stress in the tumor milieu to promote tumor progression through the inhibition of cell-mediated immunity. Indeed, in a syngeneic lymphoma model the impact of C5a in tumor microenvironment is dose-dependent (69).

Complement effectors can also affect tumor progression independently of complement activation. Factor B and factor I promote squamous cell tumor growth upon the activation of ERK1/2 (70, 71). C1q promote angiogenesis and lung metastasis in a syngeneic model of murine melanoma (72). In malignant pleural mesothelioma, C1q binds to hyaluronic acid in the tumor microenvironment and enhances tumor proliferation (73). C1q secreted by mesenchymal stromal cells mediates the activation of β-catenin in chronic lymphocytic leukemia and enhances malignant progression (74). On the other hand, properdin, a positive regulator of complement activity, induces endoplasmic reticulum-stress response and exerts a tumor suppressive role in breast cancer (75).

**Figure 1:**
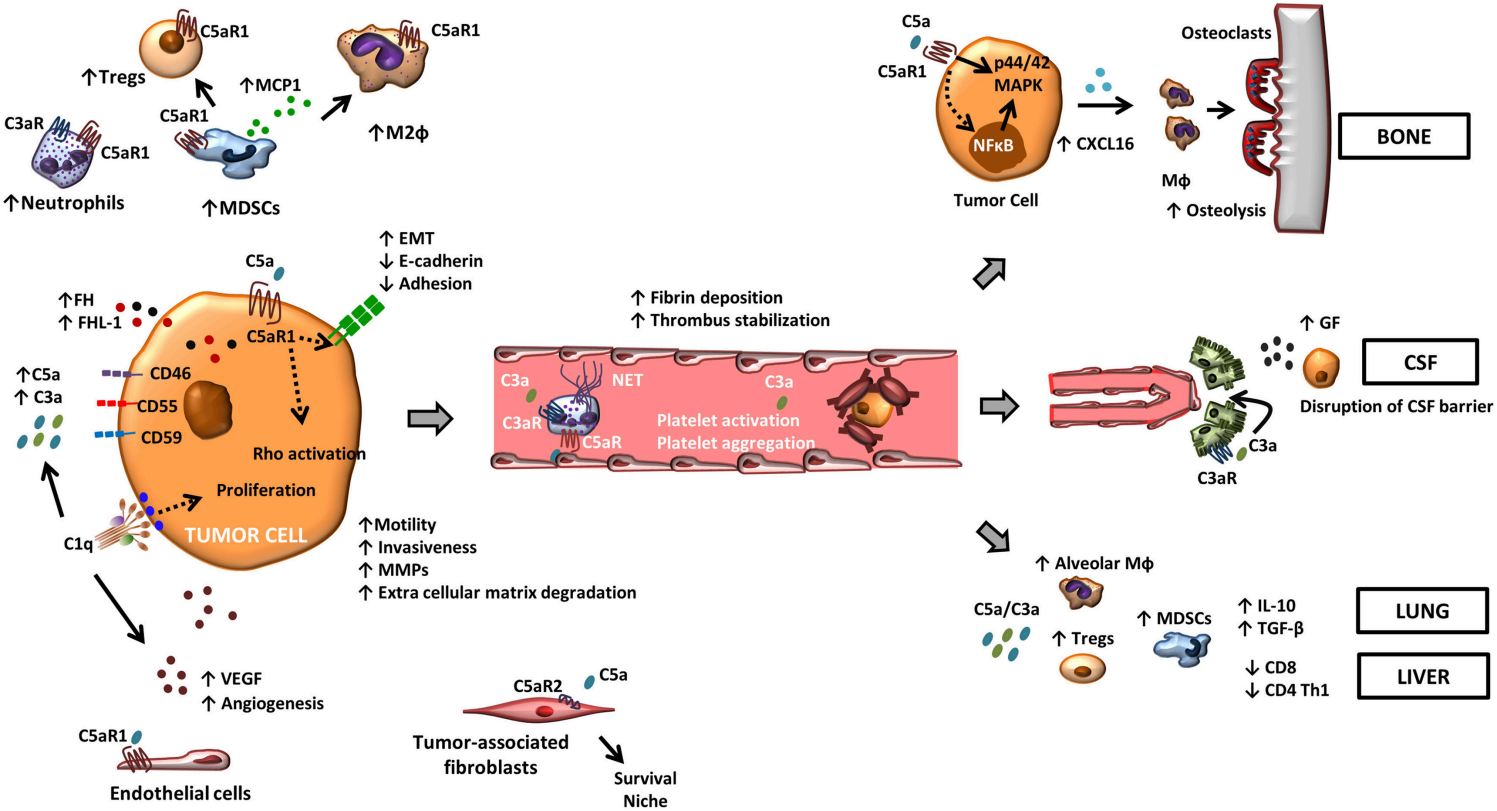
The role of complement in metastasis. Tumor-associated complement activation generates anaphylatoxins C5a and C3a in the tumor microenvironment. Binding of these molecules to their cognate receptors promote a range of tumor-promoting functions. C5a, through its receptor C5aR1, facilitates the recruitment and the activity of suppressive leukocyte subsets such as MDSCs, neutrophils, and Tregs in the tumor microenvironment. C3a also contributes to a suppressive tumor microenvironment by recruiting neutrophils. C5aR1 signaling affects endothelial function and tumor-associated angiogenesis, and the binding of C5a to C5aR2 in carcinoma-associated fibroblasts promotes tumor formation by providing a survival niche for cancer stem cells. In tumor cells, C5a/C5aR1 axis modulates tumor-induced MMP expression, increases tumor cell migration and invasiveness, enhances the release of pro-angiogenic factors, and induces EMT. Binding of C1q to tumor cells enhances tumor cell proliferation and favors angiogenesis in a complement activation-independent manner. Complement anaphylatoxins also facilitate tumor dissemination by stimulating a hyper-coagulation state and NETs, and adapt specific organ environments to the metastatic spread. This includes the disruption of the CSF barrier, the induction of CXCL16-mediated osteoclastogenesis, and the generation of an immunosuppressive microenvironment.

In summary, tumors are able to perturb complement-related immune effectors favoring tumor progression. Distorted complement homeostasis remodels the tumor microenvironment by inhibiting the anti-tumor immune responses and contributes to the metastatic dissemination of cancer cells (Figure 1).

## Early Metastasis Steps: Complement Effects on Tumor Cells

Epithelial–mesenchymal transition (EMT), loss of cell-cell adhesion, and increase of motility, invasiveness, and intravasation of tumor cells are at the core of the early metastatic events (18). Tumor-associated complement-activation modifies the tumor cell behavior endowing early metastatic traits. Perturbed complement activation leads to the generation of growth factors, proangiogenic factors, and other mediators that promote tumor growth and dissemination. These acquired pro-metastatic functions are mediated by the C3a and C5a stimulation of C3aR and C5aR1 in tumor cells, respectively, which triggers spurious intracellular signaling pathways. For instance, C5aR1 in lung tumor cells activates the p44/42 MAPK and NF-κB signaling pathways leading to the secretion of IL-8, VEGF, and MCP-1 to the tumor milieu (53). Complement components facilitate tumor dissemination by inducing an EMT in tumor cells which leads to the acquisition of a motile and less adherent phenotype. C5a/C5aR1 axis mediates the upregulation of transcription factor Snail and a concomitant decrease in E-cadherin and claudin-1 gene expression levels with increased invasiveness in hepatocellular carcinoma (76). In ovarian cells, TWIST1 enhances C3 expression and mediates EMT (77). According to these findings C5aR1-tumor expression was associated with tumor invasiveness, vascular and lymphatic invasion, liver metastasis, and poor outcome in patients with gastric tumors (78). Furthermore, C5aR1 inhibition hampers lung cancer cell migration, and up-regulates the expression of E-cadherin, suppressing EMT and invasiveness. Consistently, a negative correlation between the expression of C5aR1 and E-cadherin was found in lung primary tumors (79).

Initial steps for the acquisition of a metastatic phenotype also involves the secretion of stromelysins and other matrix metalloproteinases (MMPs) able to degrade different extracellular matrix components, especially the basal membrane, allowing for tumor cell intravasation and dissemination to local or distant sites (33). C5a markedly enhances cancer-mediated MMP activities and migratory and invasive tumor cell activities (80). C5a stimulation also decreases tumor adhesion to extracellular matrix proteins including collagens I and IV (53). Aberrantly expressed C5aR1 increases cell locomotion, cytoskeletal rearrangements with the formation of lamellipodia and membrane ruffling in liver bile duct malignant cells (80). C5aR1 signaling promotes motility and invasiveness through the activation of RhoA, and leads to enhanced invasion and vascular invasion in gastric cancer cells (56). ERK and PI3K, downstream C5aR1 activation, mediate an increase in cell invasiveness in renal cancer cells (81).

C3a-mediated stimulation elicits an increase in p42/44, p38 MAPK, and PKB/AKT activation and downregulates inducible hemeoxygenase-1 (HO-1) in leukemic cells (82). Autocrine stimulation of C5aR1 and C3aR upon C5a and C3a binding leads to PI3K/AKT signaling and regulates the proliferation and invasiveness of ovarian tumor cells (57).

In summary, complement-mediated effects are crucial in the early stages of metastasis, involving changes in tumor cell adherence to surrounding stroma and neighboring cells, increasing local invasiveness and promoting lymphatic and hematogenous dissemination.

## Complement Effects on Dissemination

The host microenvironment at local or distant sites provides signals permissive for tumor promotion. Critical pathways triggered in the surrounding stroma and/or endothelial or lymphatic cells are required for proper cell-cell and cell-matrix engagement and for the secretion of a panoply of protumorogenic factors (83, 84). In addition, vascular or lymphatic vessels provide a major route by which tumor cells exit the primary tumor site, enter the circulation and establish metastasis (85). Furthermore, tumor vascular density is a prognostic indicator of metastatic dissemination. In cancer, complement may be involved in the modulation of the angiogenic program in the tumor microenvironment, although the specific role of complement in angiogenesis is highly dependent on the tumor type. For instance, C5aR1 blockade does not affect tumor angiogenesis in murine models of lung or cervical cancer (50, 61). In contrast, genetic inhibition of C3 and C5aR1 impairs endothelial cell function in an ovarian cancer model (86). C5a also supports an angiogenic program displayed by infiltrating macrophages in squamous cell carcinoma (64). C1q deposition on melanoma cells increases tumor vascular density and facilitates tumor progression (72). The evidence that complement has a role in endothelial homeostasis might have implications also at secondary metastatic sites, a possibility which remains largely unexplored.

Once in the circulation, tumor cells have to overcome the mechanical constraints imposed by sheer-stress, anoikis induced by cell anchorage-independency, and the immune attack. A role of platelets, together with fibrin and thrombin, has been invoked for the establishment of distant metastasis by protecting circulating tumor cells from mechanical stress and facilitating engraftment at target sites (87).

Complement components contribute to a hyper-coagulation state allowing tumor cell survival in the circulation. C3a induces platelet activation and aggregation favoring a pro-thrombogenic state (88). Similarly, C5a stimulates neutrophils to release Tissue Factor, inducing a prothrombotic phenotype (89). C3aR in neutrophils stimulates neutrophils extracellular traps (NETs) (90), extracellular structures composed of chromatin and degrading enzymes (myeloperoxidase, cathepsin G, and elastase) that contribute to form a three-dimensional scaffold that supports fibrin deposition and thrombus stabilization and entraps platelets, erythrocytes and tumor cells, driving a protumorogenic state (91).

This pro-tumorogenic milieu also favors the subsequent dissemination of tumor cells to neighboring or distant sites. Homing of tumor cells to target sites could also be actively mediated by factors released by target organs that act as potent tumor cell chemoattractants (92). But tumors also precondition target organs creating a hospitable niche by the mobilization of bone marrow-derived myeloid cells, tumor secreted factors such as VEGF, TGF, TNF (93–95), and tumor released-exosomes which also modulate the tumor microenvironment (96, 97). These nanometer-sized vesicles, which contain a complex cargo of membrane receptors, nucleic acids, cytoskeletal components, and intracellular proteins, act as unique vehicles for transport to local or distant organs. Tumor derived exosomes, which are more abundantly released in inflammation, represent another mechanism of immunosuppression. As observed for tumor cells, exosomes display CD55 and CD59, conferring resistance against complement-mediated lysis (98), and potentially regulating the exosome-mediated cross-talk associated with the metastatic program.

These events largely studied in murine models collectively contribute to prepare the “fertile soil” invoked by the Paget's hypothesis (99), and crystallize the concept of “premetastatic niche” (100). The premetastatic niche consists in the accumulation of aberrant immune cells and extracellular matrix proteins in target organs (101). Emerging data demonstrate that C5a contributes to the lung premetastatic niche by regulating the expression of TGF-β and IL-10 by immature myeloid cells and the subsequent accumulation of regulatory T cells, the proliferation of resident alveolar macrophages in the premetastatic lungs, and a decrease in the number and the maturation status of lung dendritic cells. As a consequence, effector CD4 T-cell responses skew toward Th1 responses (102, 103).

## Late Steps of Metastasis

A similar paradigm to which occurs in the primary tumor could also influence metastatic behavior in the target organ. Tumor cells need to overcome the constraints imposed by the “foreign soil” and require compatibility with the hosting milieu. Each organ provides unique opportunities which could be exploited in the benefit of tumor cells by propelling the growth of micro to macrometastases (104). An increasing body of evidence indicates that complement is involved in this process, resulting in tumor outgrowths at secondary sites.

Genetic abrogation of C5aR1 in the host dampens M2-polarized tumor associated macrophages, leading to a decrease of liver and lung metastases in a syngeneic colon cancer model (52). Pharmacological inhibition of C5aR1 increases the infiltration of CD8 cytotoxic T cells in metastatic nodules, and impairs lung and liver metastatic processes with no effect detected in primary tumors. Thus, genetic or pharmacological inhibition of C5aR1 results in impaired metastasis (103).

Moreover, activation of C5aR1 in tumor cells leads to an increased prometastatic activity. For instance, in a lung cancer model of bone metastasis, C5a/C5aR1 axis induced the production of pro-osteoclastogenic factors favoring skeletal metastases. Among these factors, CXCL16 released upon C5aR1 signaling led to osteoclastogenic activation and osteolytic lesions. These effects were blocked by C5a inhibition or genetic silencing of C5aR1 in tumor cells, suggesting its implication in skeletal metastases (53). Indeed, complement is involved in bone homeostasis and turnover (105). Bone-forming osteoblasts and bone-resorbing osteoclasts are tightly regulated to ensure a balanced bone mass. Receptor activator of nuclear factor k-B ligand (RANKL), which is secreted by osteoblasts, binds to its receptor on the membrane of committed monocytes to differentiate into osteoclasts (106). Complement modulates osteoclasts differentiation *in vitro* and *in vivo* through C5aR1, but no effects were exerted in osteoblast differentiation (107). However, C3aR and C5aR1 signaling by C3a and C5a in osteoblasts modulates the release of pro-inflammatory pro-osteoclastogenic cytokines IL-6 and IL-8, and C5a increases RANKL in osteoblasts, overall favoring a pro-osteoclastogenic milieu (108). Because of these bone-specific mechanisms, the complement system might be specially relevant in skeletal metastases (53). Indeed, lung primary tumors that metastasize to bone show higher C5aR1 levels than those that metastasize to other locations, suggesting its major role in the tumor-induced skeletal lesions. Nevertheless, this axis also mediates lung metastases, since lung tumor colonization was decreased when lung cancer cells were devoid of C5aR1 (53).

In brain metastases, an elegant study by Massagué et al. unveiled a different prometastatic mechanism. C3 was upregulated in four leptomeningeal metastatic models and proved necessary for tumor growth within the leptomeningeal space. C3a, generated after C3 cleavage and bound to the C3aR expressed on the choroid plexus, was able to disrupt the blood-cerebrospinal fluid barrier. This effect was critical since blockade of this step provided a survival benefit in these models. However, C3 did not mediate cancer cell entry into the cerebrospinal fluid but other determinants were required for full tumor cell colonization (109).

Inhibition of complement-related proteins, and specially anaphylatoxins (14), has been proposed as a therapeutic option for maximizing the clinical efficacy of current immunotherapies. Recent studies have provided support of this idea after combined inhibition of anaphylatoxins and PD-1 signaling for the treatment of metastatic cancer. Administration of PD-1/PD-L1 blocking antibodies resulted in intratumoral complement activation and the subsequent accumulation of C5a within the tumor milieu (110). Importantly, the combination of C5a and PD-1 blockade reversed CD8 T-cell exhaustion, and markedly reduced lung cancer metastasis in two syngeneic animal models (111).

## Conclusions

The complement system represents an important player in tumorigenesis and metastasis. Its relevance stems from its ability to foster a protumorogenic milieu by modulating tumor-immune responses. It also endows tumor cells with cell functions required for metastatic dissemination. Preclinical studies support the idea that the therapeutic blockade of complement has potential in combinatorial immunotherapy to effectively eradicate primary tumors and distant metastases. A better understanding of the mechanisms of interaction of the complement system with tumor cells and their microenvironment is required for designing combined novel immunotherapeutic regimens able to effectively target established tumors.

## Author Contributions

DA, RP, and FL designed the concept. DA and FL wrote the manuscript. SO-E prepared the figure. All authors read and approved the final version of the manuscript.

### Conflict of Interest Statement

The authors declare that the research was conducted in the absence of any commercial or financial relationships that could be construed as a potential conflict of interest.
